# Evaluation of the efficacy and safety of TAS0313 in adults with recurrent glioblastoma

**DOI:** 10.1007/s00262-022-03184-7

**Published:** 2022-04-04

**Authors:** Yoshitaka Narita, Yoshiko Okita, Yoshiki Arakawa

**Affiliations:** 1 Department of Neurosurgery and Neuro-Oncology, National Cancer Center Hospital, 5-1-1 Tsukiji, Chuo-ku, Tokyo 104-0045 Japan; 2 Department of Neurosurgery, Osaka International Cancer Institute, Osaka, Japan; 3 Department of Neurosurgery, Osaka University Graduate School of Medicine, Osaka, Japan; 4 Department of Neurosurgery, Kyoto University Graduate School of Medicine, Kyoto, Japan

**Keywords:** Cancer vaccines, Cancer peptide vaccine, Glioblastoma, High-grade glioma, TAS0313

## Abstract

**Background:**

TAS0313 is a multi-epitope long peptide vaccine targeting several cancer-associated antigens highly expressed in multiple cancer types, including glioblastoma (GBM). This cohort of a Phase 2 part evaluated the efficacy and safety of TAS0313 in patients with GBM.

**Methods:**

TAS0313 (27 mg) was administered subcutaneously on Days 1, 8 and 15 of Cycles 1 and 2, and Day 1 of subsequent cycles in 21-day cycles. The primary endpoint was the objective response rate (ORR). The secondary endpoints were the disease control rate, progression-free survival (PFS) and 6- and 12-month progression-free survival rates (PFR) and safety. Immunological response was assessed as an exploratory endpoint.

**Results:**

The best overall response was partial response in 1 patient, and the ORR (95% CI) was 11.1% (0.3–48.2%) in the per-protocol set (*n* = 9). A further 3 patients achieved stable disease, for a disease control rate (95% CI) of 44.4% (13.7–78.8%). Median (95% CI) PFS was 1.7 (1.3–NE) months and 6- and 12-month PFRs (95% CI) were 22.2% (3.4–51.3%) each. Common (≥ 20% incidence) treatment-related adverse events (AEs) were injection site reactions (*n* = 8, 80.0%), followed by pyrexia (*n* = 7, 70.0%), and malaise, injection site erythema and injection site pruritus (*n* = 2, 20.0% each). There were no grade 4 or 5 treatment-related AEs. No deaths occurred during the study. In some patients, TAS0313 treatment was confirmed to increase cytotoxic T lymphocyte and immunoglobulin G levels compared with baseline.

**Conclusion:**

TAS0313, a multi-epitope long peptide vaccine, demonstrated promising efficacy and acceptable safety in patients with recurrent GBM.

**Clinical trial registration:**

JapicCTI-183824 (Date of registration: Jan 11, 2018)

**Supplementary Information:**

The online version contains supplementary material available at 10.1007/s00262-022-03184-7.

## Introduction

The prognosis for glioblastoma (GBM) remains dismal, with a 5-year survival rate of < 10% [1]. Despite multimodal treatment consisting of maximal safe surgical resection, followed by radiotherapy (RT) with temozolomide (TMZ), and adjuvant TMZ, [2] almost all patients experience recurrence [3]. Following recurrence, no standard of care exists and treatment options are limited [4, 5]. Bevacizumab has been shown to extend progression-free survival (PFS) in recurrent GBM (rGBM) but has failed to demonstrate a survival advantage [6, 7]. Similarly, clinical trials of several immunotherapy agents performed in the setting of rGBM have proven unsuccessful [8, 9]. Treatment needs therefore remain unmet, with even the latest immuno-oncology approaches not considered effective in rGBM.

Cancer peptide vaccines are a novel type of cancer immunotherapy that exert their antitumor effect via induction of cytotoxic T lymphocytes (CTLs) reactive to recognized cancer-associated antigens. To date, clinical studies of cancer peptide vaccines have typically used short peptides consisting of 8–10 amino acid residues, which have demonstrated partial efficacy in urothelial carcinoma and GBM [10–13]. However, despite promising preliminary results, the response rate across multiple clinical studies is only 2.9% [14] due to several factors [15–17].

TAS0313 is a novel multi-epitope long peptide vaccine cocktail comprising 3 long peptide chains (TAS0314, TAS0315 and TAS0316) that harbor 12 CTL epitope peptides. These peptides are derived from 8 cancer-associated antigens known to be highly expressed in multiple cancer types, including GBM [18].

A first-in-human phase 1/2 clinical study of TAS0313 has recently been conducted in patients with advanced solid tumors for whom no standard therapy is available. Results of the dose-finding portion of the study demonstrated promising preliminary safety and efficacy at both 9 mg and 27 mg doses [19]. We report the results from Cohort B of the study, which evaluated the efficacy and safety of TAS0313 monotherapy in patients with rGBM at the dose established in the dose-finding cohort [19].

## Materials and methods

### Study design and treatment

This phase 1/2 open-label, non-randomized, multicenter study evaluated the tolerability, safety and efficacy of TAS0313 in patients with solid tumors.

The study design consisted of 4 parts, including: a dose-finding cohort (Cohort A); an efficacy-finding cohort (Cohort B); and 2 additional cohorts, which evaluated the efficacy and safety of TAS0313 in combination with pembrolizumab in patients with urothelial carcinoma without (Cohort C1) and with (Cohort C2) prior exposure to immune checkpoint inhibitors. Data from the dose-finding cohort have been reported previously; [19] here, we present the results from the efficacy-finding cohort (Cohort B).

The primary objective of Cohort B was to evaluate the efficacy of TAS0313 monotherapy in patients with rGBM using the recommended dose (27 mg) determined in Cohort A of the phase 1/2 study. Safety was a secondary objective. Exploratory objectives included peptide-specific CTL, peptide-specific immunoglobulin G (IgG), tumor-infiltrating lymphocyte (TIL) counts, blood cytokine concentrations in plasma samples and mRNA expression levels of immunological factors and target cancer-associated antigens from archival tumor tissue samples obtained by surgical resection.

The study drug was prepared by dissolving 27 mg lyophilized TAS0313 in water, which was then mixed with an adjuvant, Montanide™ ISA 51 VG, at a ratio of 1:1 for emulsification. The study drug emulsion was administered subcutaneously near the lymph node (upper back, axillary, inguinal or abdominal) on Days 1, 8 and 15 of Cycles 1 and 2, and on Day 1 of subsequent cycles in 21-day cycles.

The study was conducted in accordance with ethical principles of the Declaration of Helsinki, the International Council for Harmonisation Harmonised Tripartite Guideline for Good Clinical Practice and Institutional Review Board regulations. All patients provided written informed consent to participate in the study.

### Patients

Patients aged between 20 and 70 years with histologically confirmed Grade IV rGBM (including gliosarcoma, giant cell glioblastoma and epithelioid glioblastoma) as per World Health Organization classification criteria [20] who had either the HLA-A*02:01, HLA-A*02:06, HLA-A*02:07, HLA-A*11:01, HLA-A*24:02, HLA-A*31:01 or HLA-A*33:03 allele type were eligible.

Additional eligibility criteria included: confirmed first or second recurrence, or disease progression (PD) following standard therapy with RT and TMZ; ≥ 1 measurable lesion based on Response Assessment in Neuro-Oncology (RANO) criteria [21] by magnetic resonance imaging (MRI); a Karnofsky Performance Scale (KPS) score of ≥ 70 at enrollment; adequate hematological (absolute neutrophil count of ≥ 1500/mm^3^; hemoglobin value of ≥ 8.0 g/dL; platelet count of ≥ 75,000/mm^3^), renal [serum creatinine ≤ 1.5× upper limit of normal (ULN) or creatinine clearance of ≥ 50 mL/min] and liver (aspartate aminotransferase and alanine aminotransferase ≤ 3× ULN, total bilirubin ≤ 1.5× ULN) function; life expectancy of ≥ 3 months.

### Outcome measures

#### Efficacy

The primary efficacy endpoint was the objective response rate [ORR; defined as the proportion of patients achieving complete response (CR) or partial response (PR)], as per RANO [21] and iRANO [22] criteria.

Secondary efficacy endpoints included the disease control rate (DCR), duration of response, PFS and PFS rate (PFR) as per RANO [21] and iRANO [22] criteria. Disease control rate was defined as the percentage of patients with a best overall response of CR, PR or SD. Duration of response was defined as the period from the date of CR or PR until the date of confirmation of PD or death due to any cause, whichever occurred first. PFS was defined as the period from the date of enrollment until the date of PD or death due to any cause, whichever occurred first. Patients without any PD event at the time of analysis or receiving subsequent treatment were censored at the date of last evaluation that confirmed absence of PD. PFR was defined as the proportion of patients without PD at 6 or 12 months after the date of enrollment (6- and 12-month PFR, respectively). Tumors were assessed using MRI scans at baseline, then every 6 weeks from Day 1 of Cycle 1.

#### Safety

Safety assessments included the incidence of AEs, including serious AEs, treatment-related AEs and laboratory variables. AEs were defined as any unfavorable or unintended sign, symptom or illness occurring in a patient enrolled in the study, irrespective of its relationship to the study drug. Treatment-related AEs were defined as any AE for which a causal relationship to the study drug could not be denied. AEs were evaluated and categorized by system organ class (SOC) and preferred term (PT) using MedDRA version 23.1, with severity graded according to the Common Terminology Criteria for Adverse Events version 4.03.

As part of the safety assessment, data were also collected on vital signs, body weight, laboratory variables and 12-lead electrocardiogram findings.

#### Biomarker analysis

Peptide-specific CTL counts and peptide-specific IgG antibody concentrations were measured from blood samples collected before TAS0313 treatment (≤ 1 week before enrollment) and at Day 22 of Cycle 2 and Cycle 3; full experimental details are provided in Supplementary Table 1. CTL and IgG induction were analyzed to evaluate the pharmacodynamics of TAS0313. CTL induction was defined as patients who had ≥ 180 spots/100,000 cells based on the difference in mean + 3× standard deviation between the negative control samples of the overall patient population.

Tumor-infiltrating CD8^+^ lymphocyte count was measured from either formalin-fixed, paraffin-embedded tumor tissue samples before TAS0313 treatment (≤ 28 days before enrollment) or archival tumor tissue samples (mandatory), and at Day 22 of Cycle 2 (optional). Tumor-infiltrating CD8^+^ T lymphocytes in formalin-fixed, paraffin-embedded tissues were evaluated by immunohistochemical staining of slides with anti-CD8 (SP57) rabbit monoclonal primary Ab (Roche Diagnostics) and Ventana iVIEW DAB Universal Kit (Roche Diagnostics) according to the manufacturer’s instructions. Two fields in hotspots with CD8^+^ T cell infiltration at the tumor site were selected and CD8^+^ cells were counted.

Cytokine concentrations were measured from plasma samples collected before TAS0313 treatment (≤ 1 week before enrollment).

mRNA expression levels of immunological factors and target cancer-associated antigens were measured from archival tumor tissue samples obtained by surgical resection by Riken Genesis using a modified nCounter PanCancer IO 360 Gene Expression Panel (nanoString) as per the manufacturer’s instructions. Measurement of TAS0313 target cancer antigens was carried out in this modified panel by adding sets of probes. Human HLA-A*03:01 cDNA and Human HLA-A*24:02:01 cDNA were provided by the RIKEN BRC through the National Bio-Resource Project of the MEXT, Japan [23].

### Statistical analysis

The primary efficacy endpoint was evaluated using the per-protocol set (PPS), which included all enrolled patients who received TAS0313 on Days 1, 8 and 15 in Cycles 1 and 2 and had ≥ 1 post-treatment response evaluation available. Secondary analysis of the primary efficacy endpoint was evaluated using the full analysis set (FAS), which comprised all enrolled patients who received ≥ 1 dose of study drug. Secondary efficacy endpoints were evaluated using the FAS and PPS. Efficacy analyses were performed using a data cutoff date of 10 Sept 2020. Safety analyses were performed using the safety analysis set, which comprised all patients who received ≥ 1 dose of study drug. The pharmacodynamic-evaluable population included all patients who received ≥ 1 dose of study drug and had relevant data (e.g., CTL, IgG, tumor-infiltrating CD8^+^ TIL) available. The pharmacogenomics-evaluable population included all patients who received ≥ 1 dose of study drug and had relevant data (e.g., cytokine concentration, mRNA expression of immunological factors and target cancer-associated antigen) available.

Baseline demographics were summarized using descriptive statistics, with the mean, standard deviation and median (min, max) calculated for continuous variables and the frequency number and proportion calculated for categorical variables. Time-to-event analyses (PFS and 6- and 12-month PFR) were summarized using the Kaplan–Meier method with 95% CI. A post hoc analysis was also conducted, in which receiver operating curves (ROC) were generated to evaluate TIL count and IgG concentration cutoffs predictive of best response, with PR and SD represented as “positive” and PD represented as “negative” responses.

The frequency of adverse events was summarized descriptively overall and for each individual event (by SOC and PT).

All statistical analyses were performed using SAS software version 9.4 (SAS Institute, Cary, NC, USA).

## Results

### Patient disposition

A total of 10 patients were enrolled into Cohort B of the study between March 12, 2019, and March 30, 2020. All (*n* = 10) patients were included in the FAS, safety analysis set, the pharmacodynamic- and pharmacogenomics-evaluable populations, respectively. Nine patients were included in the PPS.

Among the safety analysis set, 8 (80.0%) patients discontinued treatment with TAS0313, most frequently due to PD (*n* = 7, 87.5%). One patient discontinued treatment due to an AE (anaphylactoid reaction, 12.5%).

### Patient characteristics and treatment

Baseline demographic and clinical characteristics of patients in the FAS are presented in Table 1. No significant differences in baseline demographics and clinical characteristics (including sex, age, KPS, prior surgery/treatment and HLA allele) were observed between patients in the FAS and PPS (data not shown).

**Table 1:** Demographic and baseline clinical characteristics (full analysis set)

	Full analysis set (*N* = 10)
Sex, male, *n* (%)	5 (50.0)
Age, years, median (min, max)	56.5 (33.0, 69.0)
*Age, years, category*	
< 65	8 (80.0)
≥ 65	2 (20.0)
*Karnofsky performance status*	
70	1 (10.0)
80	5 (50.0)
90	4 (40.0)
*Histological type*	
Glioblastoma	8 (80.0)
Gliosarcoma	2 (20.0)
*Number of prior relapses*	
1	6 (60.0)
2	4 (40.0)
*IDH1 status*	
Mutant	1 (10.0)
Wild type	8 (80.0)
Unknown	1 (10.0)
*IDH2 status*	
Wild type	8 (80.0)
Unknown	2 (20.0)
*MGMT status*	
Methylated	4 (40.0)
Unmethylated	4 (40.0)
Unknown	2 (20.0)
*HLA-A type*	
HLA-A*02:01	3 (30.0)
HLA-A*24:02	9 (90.0)
HLA-A*31:01	3 (30.0)
HLA-A*33:03	2 (20.0)

Data presented as *n* (%) unless otherwise specified

*GBM* glioblastoma; *HLA* human leukocyte antigen; *IDH* isocitrate dehydrogenase; *MGMT* methylguanine-DNA methyltransferase

### TAS0313 treatment

The median (min, max) treatment duration was 70.0 (29, 393) days, the median (min, max) number of treatment cycles was 3.0 (2.0, 18.0), and the median (min, max) total administered dose was 189.0 (108, 594) mg.

### Efficacy

The ORR (95% CI) in the PPS (the primary endpoint) was 11.1% (0.3–48.2%). The best overall response was PR in 1 (11.1%) patient, SD in 3 (33.3%) patients and PD in 5 (55.6%) patients according to RANO. Disease control was achieved in 4 patients, and the DCR (95% CI) was 44.4% (13.7–78.8%).

The ORR (95% CI) in the FAS (secondary analysis of the primary efficacy endpoint) was 10.0% (0.3–44.5%). The best overall response was PR in 1 (10.0%) patient, SD in 4 (40.0%) patients and PD in 5 (50.0%) patients according to RANO. Disease control was achieved in 5 patients, and the DCR (95% CI) was 50.0% (18.7–81.3%).

No difference in response rates were observed in the PPS and FAS when analyzed according to iRANO criteria.

**Fig. 1:**
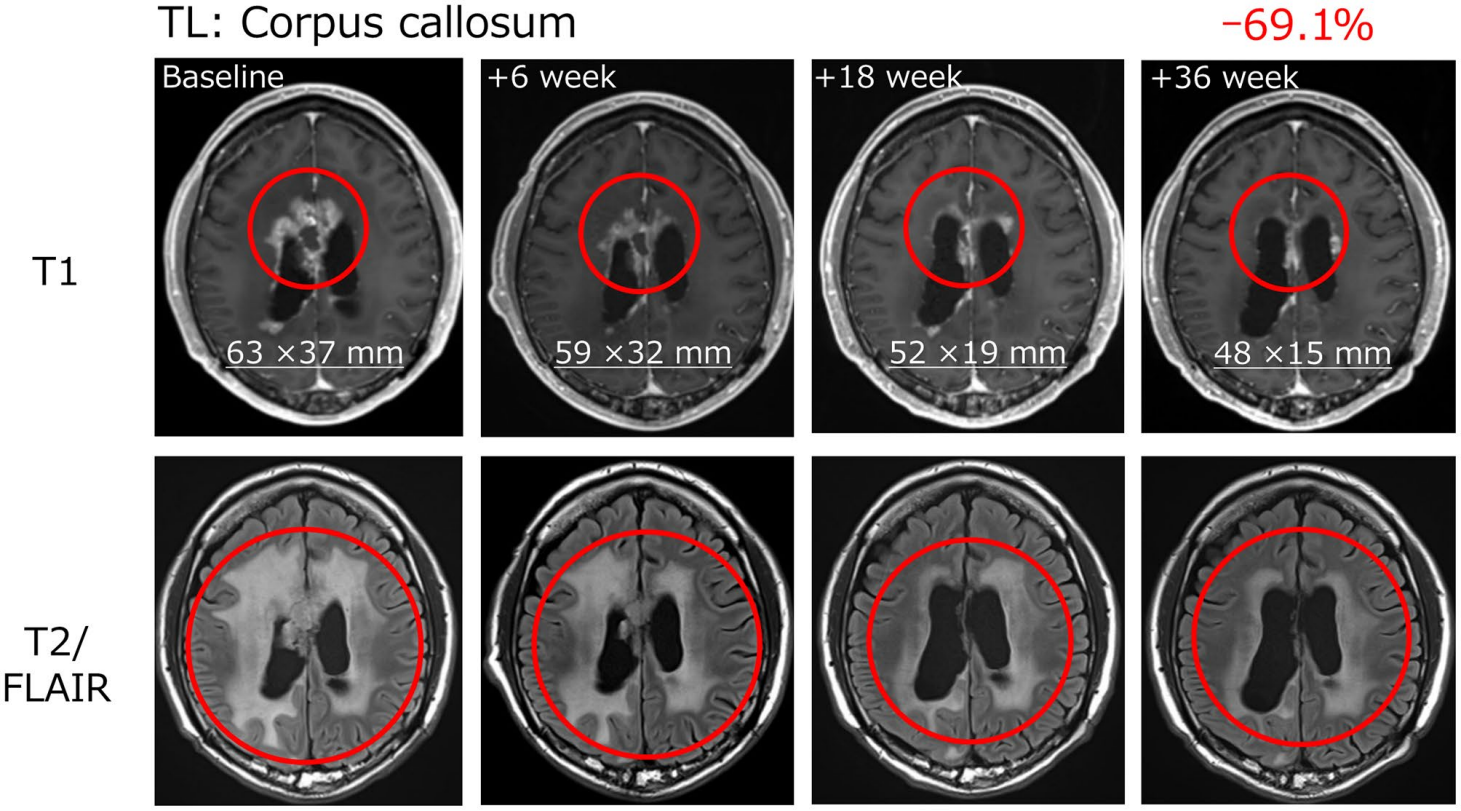
Tumor volume reduction in a patient achieving partial response with TAS0313 treatment TL, target lesion

The patient (patient number: B27-007) who achieved PR was aged 49 years, had HLA-A*24:02 allele, a KPS score of 80, MGMT promoter methylation, and was IDH wild type. The patient had failed multiple prior therapies following partial resection, including a combination of TMZ and nivolumab with/without RT, and bevacizumab and eribulin monotherapy. Following TAS0313 treatment, the patient achieved PR by Week 18, which persisted to beyond Week 36. This coincided with a reduction in tumor volume by Week 6, which reached 69.1% by Week 36 (Fig. 1).

**Fig. 2:**
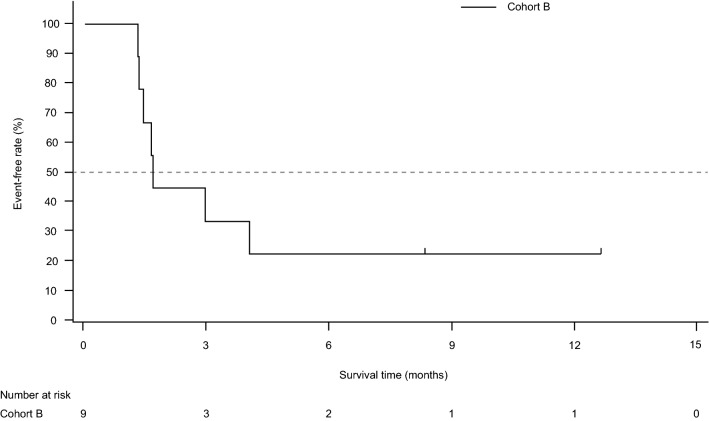
Kaplan–Meier estimate of progression-free survival in the per-protocol set

**Fig. 3:**
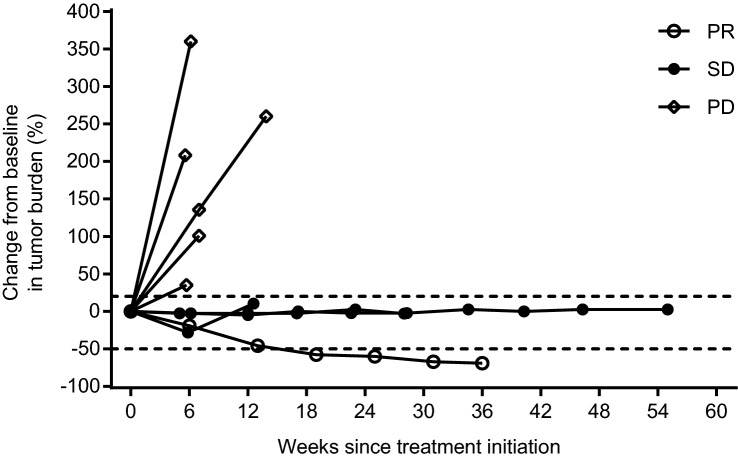
Spider plot demonstrating change from baseline in tumor burden following treatment initiation in patients treated with TAS0313 (per-protocol set)

The Kaplan–Meier estimate of PFS in the PPS is presented in Fig. 2. Median (95% CI) PFS was 1.7 (1.3–NE) months and the 6- and 12-month PFR (95% CI) was 22.2% (3.4–51.3%) at each time point. Notably, 3 patients achieved PFS of ≥ 3.5 months and 2 (22.2%) patients continued TAS0313 treatment for over 8 months (Fig. 3). The median PFS (95% CI) in the FAS was 2.3 (1.3–NE) months, and the 6- and 12-month PFR (95% CI) was 25.0% (4.1–54.8%) at each time point.

### Safety

The incidence of AEs and treatment-related AEs occurring during TAS0313 treatment are presented in Table 2.

**Table 2:** Occurrence of adverse events and treatment-related adverse events overall and by severity (safety analysis set)

	AEs		Treatment-related AEs	
	All grade	Grade ≥ 3	All grade	Grade ≥ 3
No. of cases	10 (100.0)	2 (20.0)	10 (100.0)	1 (10.0)
*Occurrence of AE/treatment-related AE (SOC/PT)*				
Cardiac disorders	1 (10.0)	0	0	0
Palpitations	1 (10.0)	0	0	0
Ear and labyrinth disorders	1 (10.0)	0	0	0
Vertigo	1 (10.0)	0	0	0
Gastrointestinal disorders	2 (20.0)	0	1 (10.0)	0
Abdominal pain	1 (10.0)	0	1 (10.0)	0
Constipation	1 (10.0)	0	0	0
Nausea	2 (20.0)	0	0	0
Vomiting	1 (10.0)	0	0	0
General disorders and administration site conditions	10 (100.0)	0	10 (100.0)	0
Injection site erythema	2 (20.0)	0	2 (20.0)	0
Injection site pain	1 (10.0)	0	1 (10.0)	0
Injection site pruritus	2 (20.0)	0	2 (20.0)	0
Injection site reaction	8 (80.0)	0	8 (80.0)	0
Malaise	2 (20.0)	0	2 (20.0)	0
Oedema peripheral	1 (10.0)	0	0	0
Pyrexia	7 (70.0)	0	7 (70.0)	0
Injection site swelling	1 (10.0)	0	1 (10.0)	0
Injection site injury	1 (10.0)	0	1 (10.0)	0
Immune system disorders	1 (10.0)	1 (10.0)	1 (10.0)	1 (10.0)
Anaphylactoid disorders	1 (10.0)	1 (10.0)	1 (10.0)	1 (10.0)
Infections and infestations	2 (20.0)	0	1 (10.0)	0
Influenza	1 (10.0)	0	0	0
Injection site abscess	1 (10.0)	0	1 (10.0)	0
Injury, poisoning and procedural complications	1 (10.0)	0	0	0
Fall	1 (10.0)	0	0	0
Investigations	2 (20.0)	0	1 (10.0)	0
Alanine aminotransferase increased	1 (10.0)	0	0	0
Blood creatine phosphokinase increased	1 (10.0)	0	0	0
Neutrophil count decreased	1 (10.0)	0	1 (10.0)	0
Musculoskeletal and connective tissue disorders	2 (20.0)	0	0	0
Arthralgia	2 (20.0)	0	0	0
Nervous system disorders	5 (50.0)	1 (10.0)	0	0
Epilepsy	1 (10.0)	1 (10.0)	0	0
Headache	2 (20.0)	0	0	0
Intracranial pressure increased	1 (10.0)	0	0	0
Seizure	1 (10.0)	0	0	0
Brain oedema	1 (10.0)	0	0	0
Psychiatric disorders	1 (10.0)	0	0	0
Insomnia	1 (10.0)	0	0	0
Renal and urinary disorders	1 (10.0)	0	0	0
Proteinuria	1 (10.0)	0	0	0
Respiratory, thoracic and mediastinal disorders	1 (10.0)	0	0	0
Oropharyngeal pain	1 (10.0)	0	0	0
Skin and subcutaneous tissue disorders	1 (10.0)	0	0	0
Hand dermatitis	1 (10.0)	0	0	0
Vascular disorders	2 (20.0)	0	1 (10.0)	0
Hot flush	1 (10.0)	0	1 (10.0)	0
Embolism	1 (10.0)	0	0	0

Data are presented as *n* (%)

*AE* adverse event; *PT* preferred term; *SOC* system organ class

A total of 10 (100.0%) patients experienced AEs during the study, 2 (20.0%) of which were grade ≥ 3 in severity. The most common treatment-related AE by PT was injection site reactions, occurring in 8 (80.0%) patients, followed by pyrexia (*n* = 7, 70.0%), and malaise, injection site erythema and injection site pruritus (*n* = 2, 20.0% each). Dermatological injection site reactions (Supplementary Table 2) occurred in 9 (90.0%) of patients and included injection site reaction (*n* = 8, 80.0%), injection site erythema (*n* = 2, 20.0%), injection site pruritus (*n* = 2, 20.0%), injection site pain (*n* = 1, 10.0%), injection site abscess (*n* = 1, 10.0%), injection site swelling (*n* = 1, 10.0%) and injection site injury (*n* = 1, 10.0%).

All treatment-related AEs were grade 1 or grade 2 in severity, with the exception of 1 (10.0%) patient, who experienced a grade ≥ 3 anaphylactoid reaction. This event resolved following appropriate treatment with electrolyte solution and antihistamines and TAS0313 discontinuation. No deaths occurred during the study. No other patients discontinued treatment due to AEs, and no patients experienced dose interruptions due to AEs.

### Biomarker analysis

**Fig. 4:**
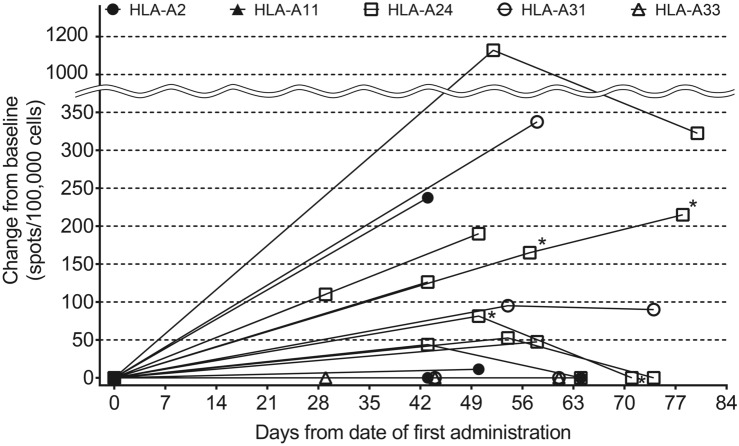
Variation in cytotoxic T lymphocyte counts following TAS0313 administration by HLA Type^a^ (pharmacogenomic-evaluable population). ^a^Days from date of first treatment = (measurement date) − (date of first administration) + 1. When change from baseline was less than zero, it was treated as zero. Patients evaluable for pharmacodynamics analysis were all treated patients who had available data on CTL, IgG or tumor-infiltrating CD8^+^ T lymphocytes. *CTL*, cytotoxic T lymphocyte; *HLA*, human leukocyte antigen; *IgG*, immunoglobulin G

We analyzed the induction of CTL and IgG to evaluate the pharmacodynamics of TAS0313. CTL induction was confirmed in 6 (60.0%) patients (Table 3). Variation in CTL counts before and after TAS0313 administration by HLA type is presented in Fig. 4.

**Table 3:** Correlation between immunological response and safety and efficacy in patients with glioblastoma treated with cancer peptide vaccine TAS0313

Patient number	HLA-A type	IgG level^a^	CTL^b^	TIL^c^	Antigen^d^	Injection site reaction (grade)	Pyrexia (grade)	Best response	PFS, months
B27-001	HLA-A*24:02; HLA-A*31:01	+	–	+	7	1	1	SD	4.0
B27-002	HLA-A*24:02	+	+	–	8	2	2	PD	1.6
B27-003	HLA-A*02:01; HLA-A*24:02	+	–	NE	7	1	1	SD	3.0
B27-004	HLA-A*24:02	+	+	+	8	1	2	SD	12.7
B27-005	HLA-A*31:01; HLA-A*33:03	+	–	–	7	1	1	PD	1.4
B27-006	HLA-A*24:02; HLA-A*31:01	+	+	–	8	1	0	PD	1.3
B27-007	HLA-A*24:02	+	+	+	8	1	0	PR	8.3
B27-008	HLA-A*02:01; HLA-A*24:02	+	+	–	8	0	2	PD	1.3
B27-009	HLA-A*24:02; HLA-A*33:03	+	–	+	8	1	1	SD	1.7
B27-010	HLA-A*02:01; HLA-A*24:02	+	+	–	8	2	0	PD	1.7

^a^At least 1 IgG level ≥ 30% compared with baseline; ^b^≥ 180 spots/100,000 cells during the study period; ^c^TIL count of ≥ 87 at baseline; ^d^Target cancer-associated antigen expression-positive number out of 8 antigens at baseline (Table S3)

*CTL* cytotoxic T lymphocytes; *HLA* human leukocyte antigen; *IgG* immunoglobulin G; *NE* not evaluable; *ORR* objective response rate; *PD* disease progression; *PFS* progression-free survival; *SD* stable disease; *TIL* tumor-infiltrating lymphocytes

CTL counts increased significantly compared with baseline in the patient with the HLA-A*24 allele type, reaching a peak of ~ 1200 spots/100,000 cells after Day 49, before declining to Day 80, although counts remained above 200 spots/100,000 cells at all time points. Although less pronounced, patients with the HLA-A*2, HLA-A*A11, HLA-A*31, HLA-A*33 alleles also experienced gradual but persistent increases in CTL counts following TAS0313 administration.

IgG induction, defined as patients with an elevation in IgG of ≥ 30% to at least 1 epitope in TAS0313 following treatment compared with baseline, was confirmed in all 10 (100.0%) patients. Induction of IgG to all peptides was observed regardless of antigen type (Supplementary Table 3).

A correlation between immunological response and efficacy was observed in 2 patients (Table 3), as evidenced by an IgG level ≥ 30% and CTL count ≥ 180 spots/100,000 cells, which correlated with prolonged PFS (ongoing at 12.7 months and 8.3 months, respectively). However, both IgG and CTL levels were not significantly higher in the 2 patients with prolonged PFS compared with the other 4 patients (Supplementary Table 3, Fig. 4). No trend was observed between injection site reaction and pyrexia severity and degree of immunological response.

To further examine the patient populations in which efficacy of TAS0313 was observed, the correlation between baseline biomarkers and efficacy was analyzed.

**Fig. 5:**
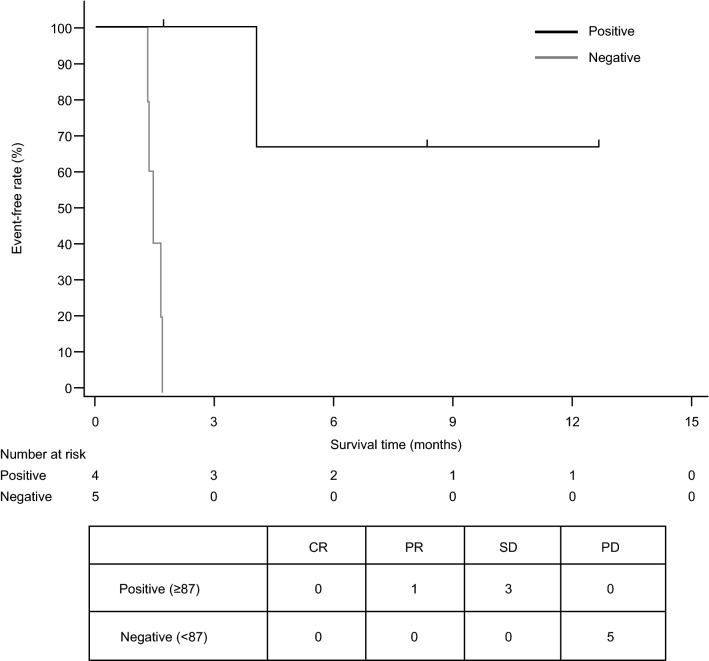
Progression-free survival in patients with recurrent glioblastoma according to tumor-infiltrating lymphocyte count (pharmacodynamic-evaluable population ^a^). ^a^Ten patients had pharmacodynamic data available and were included in this analysis. *CR*, complete response; *PD*, disease progression; *PFS*, progression-free survival; *PR*, partial response; *SD*, stable disease

For the post hoc ROC analysis, the ideal cutoff value of TIL for best overall response was calculated as 87. Patients with a TIL count ≥ 87 (*n* = 4) had prolonged PFS and superior responses [median PFS (95% CI), NR (4.0–NR); PR, *n* = 1 (25.0%); SD, *n* = 3 (75.0%)] compared with patients with a TIL count < 87 (*n* = 5) [median PFS (95% CI), 1.4 (1.3–1.7) months; PD, *n* = 5 (100.0%)] (Fig. 5). Each score of immune-related cells in pretreatment archival tumor tissue samples was measured by nCounter. In addition to CD8^+^ T cells, the population difference of many immune-related cells was observed. The score of macrophages was significantly high, the score of myeloid cells including MDSC and Treg also tended to be high in cases of PR and SD (data not shown).

**Fig. 6:**
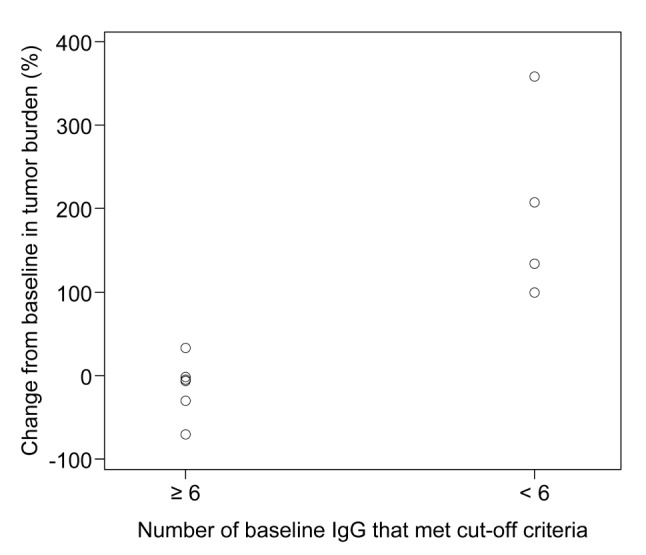
Scatter plot presenting change from baseline in tumor burden by IgG subgroup at baseline

The relationship between peptide-specific IgG concentrations of the pre-treated sample and tumor volume change (%) from baseline were also evaluated. Twelve baseline IgG cutoff values were established by applying ROC curve analysis, and all IgG concentrations were defined as having met or not met the cutoff criteria (cutoff criteria, TA1: ≥ 1748.75 pg/mL, TA2: ≥ 9710.43 pg/mL, TA4: ≤ 93,507.37 pg/mL, TA5: ≥ 6404.04 pg/mL, TA6: ≥ 3334.06 pg/mL, TA7: ≥ 33,638.80 pg/mL, TA8: ≥ 25,796.77 pg/mL, TA9: ≥ 53,945.56 pg/mL, TA10: ≥ 12,772.04 pg/mL, TA13: ≥ 4345.17 pg/mL, TA15: ≥ 2901.76 pg/mL, TA18: ≥ 1643.41 pg/mL). Of the cutoff criteria, TA5, TA6, TA8, TA9 and TA10 showed superior sensitivity and specificity (≥ 70%) (Supplementary Table 4). Interestingly, tumor shrinkage in the patient with baseline preexisting IgG to ≥ 6 peptides that met the cutoff criteria was significantly better than in the other patients, suggesting that peptide-specific IgG positivity prior to TAS0313 treatment potentially contributed to antitumor activity (Fig. 6).

Cytokine concentrations and mRNA expression of immunological factors and target cancer-associated antigens at baseline in patients receiving TAS0313 are presented in Supplementary Tables 5–8. The mRNA expression of almost all target cancer‐related antigens and HLA-A were confirmed in all enrolled patients (Supplementary Table 5). No correlation was found between mRNA expression of immunological factors within the tumor and PFS or blood cytokine concentrations and PFS, respectively (Table 3 and Supplementary Tables 5–8).

## Discussion

In this efficacy-finding cohort (Cohort B) of a phase 1/2 study, the promising efficacy of TAS0313 was shown in adult patients with rGBM. The ORR (primary endpoint) was 11.1%, and marked tumor shrinkage was confirmed in 1 patient who achieved PR. This patient achieved PR by Week 18 of TAS0313 treatment, which was maintained to beyond Week 36. MRI examination revealed a corresponding reduction in tumor volume by Week 6, with a clinically significant reduction of 69.1% by Week 36. Further, TAS0313 resulted in stabilization of disease in 3 (33.3%) patients in the PPS, for a DCR of 44.4%. The median PFS was 1.7 months, and the 6- and 12-month PFR was 22.2% at each time point. Three patients achieved PFS ≥ 3.5 months. Taken together, these findings suggest that TAS0313 has promising activity in inducing tumor shrinkage and suppressing fast-growing GBM tumor growth for an extended period of time.

The recommended dose evaluated in this cohort was 27 mg based on an absence of DLTs observed at this dose during the dose-finding cohort of this study [19]. Importantly, no major safety concerns were identified at this dose, and the safety profile was consistent with the findings reported in the dose-finding cohort of the study [19]. AEs occurred in all patients (*n* = 10, 100.0%); injection site reactions were the most common AE, occurring in 80.0% of patients, all of which were grade ≤ 2 in severity and manageable without TAS0313 discontinuation. Other common treatment-related AEs (occurring with an incidence of ≥ 20%) included pyrexia (*n *= 7, 70.0%), and malaise, injection site erythema and injection site pruritus (*n* = 2, 20.0% each). Dermatological injection site reactions, comprising injection site reaction, injection site erythema, injection site pruritus, injection site pain, injection site abscess, injection site swelling and injection site injury, occurred in 9 (90.0%) patients. One patient (10.0%) experienced a grade ≥ 3 treatment-related AE (anaphylactoid reaction), which resolved immediately without any symptoms of shock and necessitated TAS0313 discontinuation. No deaths occurred during the study.

Treatment options following rGBM remain challenging. Bevacizumab was approved by the Food and Drug Administration in 2017 for the treatment of rGBM on the basis of encouraging phase 1/2 data, [6] but subsequent studies failed to demonstrate prolongation of OS, and unmet needs remain [7]. The promising preliminary efficacy and favorable safety profile observed in the current study suggest that TAS0313 may present a more attractive alternative therapy prior to bevacizumab. Notably, the efficacy of this vaccine was comparable with, or superior to, other peptide vaccines for GBM [24, 25]. However, further investigation aimed at identifying those patients most likely to derive greatest benefit from TAS0313 is warranted.

Consistent with the findings from the dose-finding portion of the study, [19] treatment with TAS0313 27 mg was associated with induction of CTL and IgGs compared with pretreatment, thus confirming the immune activation effects of TAS0313. Although the increase in CTL counts was most pronounced in the patient with the HLA-A*24 allele type (peak of ~ 1200 spots/100,000 cells after Day 49), patients with other alleles also experienced gradual but persistent increases in CTL counts following TAS0313 administration. These findings suggest that TAS0313 may be an effective treatment for multiple HLA phenotypes.

In patients with positive CTL and IgG in our study, long-term PFS was observed in 1 patient with PR (B27-007) and 1 patient with SD (B27-004) (Table 3). This is in contrast to the dose-finding portion of the study, [19] which found no clear relationship between immune and clinical responses. Changes in TIL counts after treatment could not be evaluated because biopsy of the tumor specimen could not be performed.

A TIL count of ≥ 87 at baseline appeared to be predictive of prolonged PFS and higher DCR in the post hoc analysis (Fig. 5). A correlation between TIL and PFS was observed in patients with rGBM in our study, which is consistent with the results reported in studies with other immunotherapy agents [26, 27]. Specifically, patients in the group with a high TIL count before TAS0313 vaccination experiencing significantly prolonged PFS versus those with a low TIL count. In contrast, TIL was not associated with prolonged survival in patients not receiving immunotherapy in similar studies in GBM [27]. Tumors with low TIL counts are recognized as having an immunosuppressive microenvironment that excludes immune cells to around the tumor and prevents them from infiltrating the tumor microenvironment [28]. Although the exact reason why TIL count correlates with prognosis is unclear, tumors with preexisting lymphocytic infiltration can be expected to have a favorable microenvironment for the immune system. Therefore, in tumors with high TIL counts, CTL induced by TAS0313 administration may efficiently migrate into the tumor and exert effector activity. The conditions for CTL, IgG, TIL and antigen expression were optimal and consistent with the known mechanism of action of TAS0313 in the 2 patients with prolonged PFS. No clear relationship was found between cytokine profile or mRNA expressions of immunological factors and prognosis.

The appropriate peptides for each patient in the ITK-1 study were chosen based on the peptide-specific IgG titer at baseline, expecting activation of secondary immune responses against tumor antigen [25]. On the other hand, it has been reported that even if IgG to the peptide cannot be detected before treatment, if IgG is induced after treatment, antitumor effects can be obtained [29]. The relationship between IgG antibody titers before treatment and the clinical effectiveness of peptide vaccines has been variously reported and remains unclear, but findings from the current study suggest that peptide-specific IgG concentrations at baseline were a predictive marker of tumor shrinkage. Since TAS0313 included several ITK-1 peptides, stratification of patients with high peptide-specific IgG concentration prior to treatment is likely to lead to better outcomes in future clinical trials. In addition, SART2 (TA5), SART3 (TA6, TA9) and Lck (TA8, TA10) demonstrated excellent sensitivity and specificity among the cutoff criteria for each IgG set in this study and may be independent predictors of efficacy. Conversely, as shown in Fig. 6, the more IgG types that met the cutoff criteria, the better the antitumor effect. However, further study is warranted.

This study also had some limitations that are inherent to phase 1/2 studies and must be considered. The sample size was small (*n* = 10) and the study was conducted in Japanese patients. Therefore, extrapolation of these results to other patient populations should be made with caution. Further, an independent control arm was not included as part of this study due to the relative rarity of GBM, making comparisons difficult. A comparative study is therefore warranted in future and is necessary to confirm that TIL and IgG are not prognostic predictors but rather markers of TAS0313 efficacy.

Nevertheless, the results of the efficacy-finding portion of this phase 1/2 study demonstrated the promising efficacy and manageable safety of TAS0313, a multi-epitope long peptide vaccine targeting several cancer-associated antigens, in adult patients with rGBM, a patient population with extremely poor prognosis and limited treatment options. The promising efficacy and manageable safety findings observed in this study support the further study of TAS0313 in patients with rGBM.

## Supplementary Information

Below is the link to the electronic supplementary material.

Supplementary file1 (PDF 373 KB)

## Data Availability

Data will not be shared according to the Sponsor policy on data sharing.

## Abbreviations

AE: Adverse event
CR: Complete response
CTCAE: Common terminology criteria for adverse events
CTL: Cytotoxic T lymphocyte
DCR: Disease control rate
DLT: Dose-limiting toxicity
DOR: Duration of response
EGFR-vIII: Epidermal growth factor receptor variant III
FAS: Full analysis set
GBM: Glioblastoma
HLA: Human leukocyte antigen
IDH: Isocitrate dehydrogenase
IgG: Immunoglobulin G
iRANO: Immunotherapy response assessment in neuro-oncology
KPS: Karnofsky performance scale
MGMT: O6-Methylguanine-DNA methyltransferase
MRI: Magnetic resonance imaging
mRNA: Messenger ribonucleic acid
ORR: Objective response rate
OS: Overall survival
PD: Disease progression
PFR: Progression-free survival rate
PFS: Progression-free survival
PPS: Per-protocol set
PR: Partial response
PT: Preferred term
RANO: Response assessment in neuro-oncology
rGBM: Recurrent GBM
ROC: Receiver operating curve
RT: Radiotherapy
SD: Stable disease
SOC: System organ class
TIL: Tumor-infiltrating lymphocyte
TL: Target lesion
TMZ: Temozolomide
ULN: Upper limit of normal
WHO: World Health Organization
