# Supplementary material for: Evaluation of the efficacy and safety of TAS0313 in adults with recurrent glioblastoma
Source: Cancer Immunol Immunother. 2022 Apr 4;71(11):2703–15. doi: 10.1007/s00262-022-03184-7 (PMC9519730; doi:10.1007/s00262-022-03184-7)
Supplement: Supplementary file 1 — Supplementary file1 (PDF 373 KB) [file 262_2022_3184_MOESM1_ESM.pdf]

Supplementary Material

Evaluation	Method
Cytotoxic T cells (CTLs) were counted by SRL Laboratories (Kanagawa, Japan) using an enzyme-linked immune absorbent spot kit (ELISpot[PLUS] for Human IFN-γ; Mabtech AB, Stockholm, Sweden) according to the manufacturer's instructions.	• Peripheral blood mononuclear cells (PBMC, 100,000 cells/well) from samples were co-cultured with peptide (10 µg/mL) corresponding to HLA type (HIV SLYNTYATL, HIV RLRDLLLIVTR, HIV RYLRQQLLGI [negative control]; LCK p246-254, WHSC2 (known as NELFA) p103-111, SART3 p302-310, SART2 (known as DSE) p93-101, SART3 p109-118, MRP3 p503-511, LCK p488-497, SART3 p734-742, LCK p90-99, TMEM189 p43-51 (also referred to as KUA), EGFR p800–809, and PTHRP p102-111; Toray Research Center, Inc., Kanagawa, Japan) in 96-well plates for 6 days.<br>• Peptide-stimulated PBMC (20,000 cells/well), peptide (10 µg/mL), and antigen-presenting (T2[174xCEM.T2]) cells (10,000 cells/well) from ATCC, Manassas, VA, USA; C1R-A0301, and C1R-A2402 from Taiho Pharmaceutical Co., Ltd, Japan[a]; and C1R-A1101, C1R-A3101, and C1R-A3303 from Kurume University, Kurume, Japan were co-cultured overnight (18 ± 0.5 hours) on an ELISpot plate.<br>• After washing, an antibody detection solution, alkaline phosphatase, and substrate solution were added sequentially, and the number of spots was analyzed by an immunospot analyzer.

	• The mean number of spots, and the coefficient of variation (%) were calculated.
IgG was evaluated by Sekisui Medical Co., Ltd. (Ibaraki, Japan) using a Bio-Plex Pro magnetic COOH bead multiplex fluoroimmunoassay (Bio-Rad Laboratories, Inc., Hercules, CA, USA) according to the manufacturer's instructions.	• Peptides (LCK p246-254, WHSC2 (known as NELFA) p103-111, SART3 p302-310, SART2 (known as DSE) p93-101, SART3 p109-118, MRP3 p503-511, LCK p488-497, SART3 p734-742, LCK p90-99, TMEM189 p43-51, EGFR p800–809, and PTHRP p102-111 from American Peptide Co., Inc., Sunnyvale, CA, USA; TAS0314, TAS0315 and TAS0316 acetate from Bachem, Bubendorf, Switzerland) were attached to Bio-Plex Pro magnetic COOH beads.<br>• The peptide-bound beads were added to diluted plasma and incubated for 60 minutes.<br>• IgG antibodies to individual peptides were then measured by a Luminexassay (Luminex Corporation, Austin, TX, USA) after reacting to a biotinylated antibody (biotinylated anti-human IgG, from Vector Laboratories, Inc., Burlingame, CA, USA).

Supplementary Table 1 Experimental Procedures for CTL and IgG Evaluation.

EGFR, epidermal growth factor receptor; LCK, tyrosine-protein kinase LCK; MRP3, multidrug resistance-associated protein 3 (MRP3); PTHRP, parathyroid hormone-related protein; SART, squamous cell carcinoma antigen recognized by T-cells; TMEM189, transmembrane protein 189; WHSC2, Wolf–Hirschhorn syndrome candidate 2 protein.

[a]The parent cell line (C1R) was supplied by ATCC, and Taiho established the stable expression strains HLA-A*03:01 and A*24:02. Human HLA-A*03:01 cDNA and Human HLA-A*24:02:01 cDNA were provided by the RIKEN BRC through the National Bio-Resource Project of the

MEXT, Japan (1).

1. Akatsuka Y, Goldberg TA, Kondo A, *et al* (2002). Efficient cloning and expression of HLA class I cDNA in human B-lymphoblastoid cell lines. Tissue Antigens. 59:502–11.

**Adverse Events**	**All Grade**	**Grade ≥3**	**Grade**				
			**1**	**2**	**3**	**4**	**5**
Dermatological injection site reactions	9 (90.0)	0	7 (70.0)	2 (20.0)	0	0	0
Injection site abscess	1 (10.0)	0	0	1 (10.0)	0	0	0
Injection site erythema	2 (20.0)	0	2 (20.0)	0	0	0	0
Injection site pain	1 (10.0)	0	0	1 (10.0)	0	0	0
Injection site pruritus	2 (20.0)	0	1 (10.0)	1 (10.0)	0	0	0
Injection site reaction	8 (80.0)	0	7 (70.0)	1 (10.0)	0	0	0
Injection site swelling	1 (10.0)	0	1 (10.0)	0	0	0	0
Injection site injury	1 (10.0)	0	0	1 (10.0)	0	0	0

Supplementary Table 2 Treatment-Related Adverse Events Associated with Dermatological Injection Site Reactions in Patients with Glioblastoma Treated with Cancer Peptide Vaccine TAS0313

Pt No.	Cycle	Specific IgG (pg/mL)														
		**TA1**	**TA2**	**TA4**	**TA5**	**TA6**	**TA7**	**TA8**	**TA9**	**TA10**	**TA13**	**TA15**	**TA18**	**TAS0314**	**TAS0315**	**TAS0316**
B-27-001	BL	4427.33	23447.44	93507.37	8103.23	4084.7	33638.8	42606.1	113976.4	27129.2	7587.52	16371.01	<27.34	20390.43	<384.38	250625.9
	2	**17683.39[a]**	**293029862.74[a]**	**72660478.56[a]**	**2222995.72[a]**	**41850.11[a]**	**357564.96[a]**	**61065.21[a]**	**4756763.03[a]**	**22522640.87[a]**	**40204.69[a]**	**1235797.34[a]**	**2065.22[a]**	**110085538.94[a]**	**674213282.21[a]**	**294223930.82[a]**
	3	**35180.21[a]**	**227214781.66[a]**	**179722333.69[a]**	**3199732.84[a]**	**67730.35[a]**	**207619.64[a]**	**65509.25[a]**	**15860489.40[a]**	**34044487.26[a]**	**41800.30[a]**	**1962348.10[a]**	**29716.06[a]**	**259111398.52[a]**	**1023615471.38[a]**	**421030115.90[a]**
B-27-002	BL	<59.77	20677.97	122716.03	4911.45	1179.34	<566.41	17043.46	49902.32	12402.41	<73.44	3847.1	<27.34	50754.26	<384.38	2494473.69
	2	**14000.66[a]**	**94124406.21[a]**	**428870188.85[a]**	**3892148.06[a]**	**4542.21[a]**	**206145.58[a]**	**132933.20[a]**	**1376494.16[a]**	**23752653.86[a]**	**91404.40[a]**	**1172747.66[a]**	**12352.98[a]**	**281676228.42[a]**	**579368118.28[a]**	**507683388.00[a]**
	3	**40547.28[a]**	**333128369.42[a]**	**412319472.61[a]**	**14199532.21[a]**	**5894.02[a]**	**374161.95[a]**	**286356.67[a]**	**2738351.79[a]**	**30875319.95[a]**	**151382.78[a]**	**6261681.96[a]**	**25049.12[a]**	**367541709.51[a]**	**1464496534.27[a]**	**1347227690.54[a]**
B-27-003	BL	<59.77	11699.83	21261.17	10145.34	8700.19	<566.41	41424.1	155186	23616.69	5848.56	8651.12	<27.34	15946.87	102842.38	98594.15
	2	**5595.81[a]**	**90234695.07[a]**	**258382478.85[a]**	**7361912.69[a]**	**51327.74[a]**	**91045.58[a]**	**134246.02[a]**	**5300254.03[a]**	**460543.14[a]**	**317033.69[a]**	**1130823.28[a]**	**6229.38[a]**	**313063662.87[a]**	**379209099.92[a]**	**284269574.08[a]**
	3	**3978.70[a]**	**81250188.87[a]**	**318657372.74[a]**	**6451632.29[a]**	**49427.44[a]**	**217480.82[a]**	**193977.49[a]**	**13839914.45[a]**	**730889.40[a]**	**91728.56[a]**	**1079607.95[a]**	**6933.60[a]**	**391441891.26[a]**	**345002415.23[a]**	**250818380.19[a]**

B-27-004[b]	BL	1748.75	88524.04	78241.15	6404.04	3334.06	<566.41	18346.45	53945.56	14987.35	<73.44	2901.76	<27.34	65756.3	909199.88	2193715.23
	2	**12442.28**[a]	**44302450.99**[a]	**31717488.56**[a]	**7721543.10**[a]	**39929.43**[a]	**1152732.84**[a]	**102104.59**[a]	**390434786.78**[a]	**893409.80**[a]	**1188087.88**[a]	**1024818.28**[a]	**241548.67**[a]	**60707159.09**[a]	**3303326135.31**[a]	**821249835.83**[a]
	3	**23031.33**[a]	**75638381.45**[a]	**103768701.25**[a]	**8949753.73**[a]	**44284.11**[a]	**2555365.20**[a]	**262952.57**[a]	**351541202.56**[a]	**1996874.56**[a]	**767881.03**[a]	**1458778.56**[a]	**358443.78**[a]	**121866676.12**[a]	**2371303487.24**[a]	**648159876.56**[a]
B-27-005	BL	<59.77	<45.31	3224.37	4846.55	2908.5	<566.41	24198.69	<1922.66	<59.77	6051.74	4056.97	<27.34	<31.64	<384.38	214142.24
	2	<59.77	**163478728.12**[a]	**248934725.87**[a]	**3620417.42**[a]	**10592.80**[a]	<566.41	**40880.39**[a]	**3140433.15**[a]	**199275.69**[a]	**15368.66**[a]	**268168.88**[a]	**3501.95**[a]	**285893972.27**[a]	**897300049.46**[a]	**393142173.83**[a]
		**2289.82**[a]	**59883330.73**[a]	**261539512.41**[a]	**3748165.93**[a]	**10506.78**[a]	**21722.92**[a]	**42602.55**[a]	**7068446.74**[a]	**705600.77**[a]	7765.07	**482716.30**[a]	**6598.47**[a]	**202498139.86**[a]	**307456061.19**[a]	**163783333.66**[a]
B-27-006	BL	<59.77	<45.31	2214.57	5747.48	1289.46	<566.41	21138.4	<1922.66	<59.77	<73.44	<39.84	<27.34	3762.26	<384.38	173760.52
	2	**2006.86**[a]	**41080235.34**[a]	**87990729.34**[a]	**637163.17**[a]	**3829.26**[a]	<566.41	**33671.03**[a]	**4022531.36**[a]	**22502133.84**[a]	**18204.37**[a]	**622822.05**[a]	**4344.93**[a]	**67521532.92**[a]	**404163081.18**[a]	**92556482.22**[a]
B-27-007[b]	BL	<59.77	9710.43	251141	6540.99	3721.88	<566.41	27750.49	<1922.66	19740.44	4660.33	3399.72	<27.34	143798.48	<384.38	276737.09
	2	**45933.25**[a]	**633097209.15**[a]	**681930681.58**[a]	**13798251.56**[a]	**8081.80**[a]	**202478.99**[a]	**43252.43**[a]	**25535546.97**[a]	**3569997.50**[a]	**25987.24**[a]	**2029894.63**[a]	**34833.72**[a]	**742252935.33**[a]	**1519071205.51**[a]	**1536347793.35**[a]

	3	**37243.23[a]**	**570838422.83[a]**	**785674030.99[a]**	**13804909.59[a]**	**10231.40[a]**	**566332.98[a]**	**87043.53[a]**	**51083187.27[a]**	**4741747.54[a]**	**42155.61[a]**	**2893171.99[a]**	**92624.37[a]**	**758876072.85[a]**	**1012470714.88[a]**	**1387956738.61[a]**
B-27-008	BL	1835.41	12850.88	8528.59	7890.14	5020.99	<566.41	30441.6	<1922.66	20906.94	5645.41	11982.21	<27.34	4802.7	<384.38	83360.78
	2	**4314.58[a]**	**55453008.01[a]**	**326563406.32[a]**	**838805.86[a]**	5127.11	**315529.87[a]**	**76875.01[a]**	**1151594.30[a]**	**96517.19[a]**	**14983.50[a]**	**245741.05[a]**	**2598.39[a]**	**273515690.79[a]**	**170512081.10[a]**	**274375627.09[a]**
B-27-009	BL	<59.77	13496.79	44805.28	6997.33	4138.42	<566.41	25796.77	8232880.82	12772.04	4345.17	<39.84	1643.41	29306.72	<384.38	153521.37
	2	**5022.40[a]**	**11640111.19[a]**	**120086589.09[a]**	**701854.62[a]**	**5912.40[a]**	**158450.71[a]**	**47258.97[a]**	**11568125.16[a]**	**805571.81[a]**	**71850.99[a]**	**688382.74[a]**	**6116.23[a]**	**131324615.35[a]**	**278810548.13[a]**	**245928236.01[a]**
B-27-010	BL	<59.77	<45.31	425430.91	4126.93	1649.92	<566.41	16026.72	<1922.66	<59.77	4135.48	2728.44	<27.34	159854.68	<384.38	90744.86
	2	**4002.90[a]**	**155730345.16[a]**	**263971313.98[a]**	**2751251.49[a]**	**53447.16[a]**	**241774.33[a]**	**54092.21[a]**	**4602223.75[a]**	**214125.40[a]**	**69727.20[a]**	**1748710.82[a]**	**1972.26[a]**	**231233811.99[a]**	**219247692.80[a]**	**532125344.98[a]**

Supplementary Table 3 Induction of Specific IgG Levels in Patients with Glioblastoma Treated with Cancer Peptide Vaccine TAS0313.

BL, baseline; IgG, immunoglobulin G; PFS, progression-free survival.

[a]≥30% increase from baseline; [b]Patients achieved long-term PFS.

IgG Antibody	Antigen	Cut-off Criteria (pg/mL)	Sensitivity	Specificity
TA1	Lck	≥1748.75	0.400	0.800
TA2	WHSC2	≥9710.43	1.000	0.600
TA4	SART3	≤93507.37	0.800	0.400
**TA5**	**SART2**	**≥6404.04**	**1.000**	**0.800**
**TA6**	**SART3**	**≥3334.06**	**1.000**	**0.800**
TA7	MRP3	≥33638.80	0.200	1.000
**TA8**	**Lck**	**≥25796.77**	**0.800**	**0.800**
**TA9**	**SART3**	**≥53945.56**	**0.800**	**1.000**
**TA10**	**Lck**	**≥12772.04**	**1.000**	**0.800**
TA13	TMEM189	≥4345.17	0.800	0.600

Supplementary Table 4 Sensitivity and Specificity of IgG Cutoff Criteria.

Pt No.	MRP3	SART2	EGFR	LCK	PTHRP	SART3	KUA	WHSC2	HLA-A	3 × standard deviation of negative control
B27-001	1748.94	790.47	448.04	19.20	61.61	2315.39	608.85	844.87	6209.31	26.043
B27-002	860.26	536.54	36824.04	13.05	163.08	3246.16	604.22	746.10	9073.10	11.973
B27-003	111.22	711.79	1878.84	11.86	65.25	1288.65	521.98	381.11	2122.04	17.639
B27-004	1325.23	1785.73	687.18	50.73	170.41	2301.71	847.29	657.06	6923.35	11.536
B27-005	212.70	529.06	5316.72	7.21	53.18	2391.13	420.90	688.59	1968.42	12.466
B27-006	1560.86	182.56	22109.59	8.46	85.84	893.48	205.54	301.05	4194.14	5.768
B27-007	3538.45	675.20	117.43	70.46	62.63	1225.15	520.59	393.38	2595.12	13.677
B27-008	2551.57	764.08	2088.49	9.26	344.99	2213.52	571.90	588.11	4322.85	9.071
B27-009	565.84	1016.10	820.14	28.62	110.09	1201.04	592.26	345.67	1412.41	9.613
B27-010	306.07	379.42	139.12	12.65	32.88	1350.74	561.54	179.59	2286.65	6.414

Supplementary Table 5 Baseline mRNA Expression Level of Immunological Factors and Target Cancer-Associated Antigens in Patients with Glioblastoma Treated with Cancer Peptide Vaccine TAS0313

EGFR, epidermal growth factor receptor; HLA, human leukocyte antigen; KUA, transmembrane protein 189 LCK, tyrosine-protein kinase LCK; mRNA, messenger ribonucleic acid; MRP3, multidrug resistance-associated protein 3; PTHRP, parathyroid hormone-related protein; SART2, squamous cell carcinoma antigen recognized by T-cells 2; SART3, squamous cell carcinoma antigen recognized by T-cells 3; WHSC2, Wolf-Hirschhorn Syndrome-Associated Genes.

Pt No.	IL-1β	IL-1RA	IL-2	IL-4	IL-5	IL-6	IL-7	IL-8	IL-9	IL-10
B27-001	1.45	87.14	<7.71	1.93	<19.99	<1.68	19.60	6.81	314.98	<5.59
B27-002	1.67	86.05	<7.62	1.99	<19.45	<1.61	18.13	7.18	334.08	<5.39
B27-005	1.54	<152.80	<7.62	1.48	<19.45	<1.61	11.93	<3.76	286.69	<5.39
B27-006	1.11	<152.80	<7.62	1.94	<21.04	<1.61	17.15	6.71	317.17	<5.39
B27-007	1.89	94.63	<7.62	1.67	<19.45	<1.61	15.09	4.62	319.22	<5.39
B27-008	1.79	97.15	<7.71	2.02	<19.99	1.94	22.21	6.81	323.07	<5.59
B27-009	1.93	188.11	<7.71	1.63	<19.99	2.25	15.41	5.62	286.50	<5.59
B27-010	2.90	102.85	<7.62	1.54	<19.45	<1.61	26.14	4.54	310.64	<5.39

Supplementary Table 6 Baseline Cytokine Levels (pg/mL) in Patients with Glioblastoma Treated with Cancer Peptide Vaccine TAS0313

IL, interleukin; RA, receptor antagonist.

Pt No.	IL-12p70	IL-13	IL-15	IL-17A	Eotaxin	Basic FGF	G-CSF	GM-CSF	IFN-γ	IP-10
B27-001	<6.82	<1.31	<37.14	16.43	53.34	38.73	41.56	<1.85	<5.15	497.61
B27-002	<6.66	<1.28	<203.37	17.61	62.25	37.88	38.14	<1.84	<5.06	293.52
B27-005	<6.66	<1.28	<203.37	14.63	44.87	31.38	26.95	<1.84	<5.06	185.36
B27-006	<6.66	<1.28	<203.55	19.26	54.19	45.99	48.23	<1.78	5.24	999.13
B27-007	<6.66	<1.28	<203.37	16.26	41.97	35.91	31.97	<1.84	<5.06	325.31
B27-008	<6.82	<1.31	<37.14	18.52	56.90	39.09	36.27	<1.85	<5.15	391.31
B27-009	<6.82	<1.31	<37.14	17.33	36.10	37.30	39.98	<1.85	5.38	273.12
B27-010	<6.66	<1.28	<203.37	15.44	40.28	37.23	<25.26	<1.84	<5.06	326.74

Supplementary Table 7 Baseline Cytokine Levels (pg/mL) in Patients with Glioblastoma Treated with Cancer Peptide Vaccine TAS0313

FGF, fibroblast growth factor; G-CSF, granulocyte colony stimulating factor; GM-CSF, granulocyte-macrophage colony stimulating factor; IL, interleukin; IP-10, interferon gamma-induced protein 10.

Pt No.	MCP-1	MIP-1α	PDGF-BB	MIP-1β	RANTES	TNF-α	VEGF
B27-001	12.88	<1.37	1014.87	253.32	5709.50	65.31	<108.63
B27-002	10.03	<1.39	732.04	226.74	5815.62	56.96	<18.61
B27-005	7.87	<1.39	478.83	213.48	5065.51	48.75	<18.61
B27-006	14.27	<1.38	505.60	251.79	6109.02	78.96	<93.88
B27-007	7.74	<1.39	1409.06	239.44	6341.34	55.57	<18.61
B27-008	16.27	<1.37	1018.16	236.27	6927.21	76.45	<108.63
B27-009	20.58	<1.37	1582.27	234.37	5252.00	66.31	<108.63
B27-010	9.06	<1.39	764.60	206.41	6467.89	52.17	<18.61

Supplementary Table 8 Baseline Cytokine Levels (pg/mL) in Patients with Glioblastoma Treated with Cancer Peptide Vaccine TAS0313
MIP-1α, macrophage inflammatory protein-1; PDGF, platelet-derived growth factor-BB; RANTES, Regulated upon Activation, Normal T Cell Expressed and Presumably Secreted; TNF, tumor necrosis factor; VEGF, vascular endothelial growth factor.
